# Aggregate index of systemic inflammation and glaucoma status: population-based evidence with clinical validation

**DOI:** 10.3389/fmed.2026.1923522

**Published:** 2026-08-27

**Authors:** Songxin Yang, Li Li, Xianming Ouyang, Rui Zhang, Gang Lin, Sunkun Pan, Guiquan Xie, Zhen Lv, Dieran Huang, Guoyan Luo, Kunpeng Pang

**Affiliations:** 1 Department of Ophthalmology, The People's Hospital of the Qiandongnan Miao and Dong Autonomous Prefecture, Kaili, Guizhou, China; 2 Department of Ophthalmology, Taijiang County People's Hospital, Taijiang, Guizhou, China; 3 Department of Ophthalmology, Qilu Hospital of Shandong University, Jinan, Shandong, China

**Keywords:** aggregate index of systemic inflammation, clinical validation, glaucoma, National Health and Nutrition Examination Survey, systemic inflammation

## Abstract

**Objective:**

Glaucoma is a major cause of irreversible visual impairment, but evidence on the association between the aggregate index of systemic inflammation (AISI) and glaucoma remains limited, particularly with clinical validation.

**Methods:**

We analyzed National Health and Nutrition Examination Survey (NHANES) data from participants aged 40 years and older. Weighted multivariable logistic regression models were used to examine the association between AISI and glaucoma. The primary outcome was composite glaucoma, defined as the presence of either self-reported glaucoma or examination-based glaucoma; the two definitions were also analyzed separately in sensitivity analyses. Additional sensitivity analyses excluded participants with extreme AISI values and, separately, participants with a white blood cell (WBC) count >10 × 10^9^/L. We also compared AISI with related blood-cell-derived inflammatory indices and applied false discovery rate correction for multiple comparisons. Finally, the main findings were validated using a hospital-based clinical sample.

**Results:**

The primary analysis included 5,455 participants, among whom 431 had composite glaucoma. After full adjustment, each doubling of AISI was associated with higher odds of composite glaucoma (OR = 1.20, 95% CI: 1.05–1.37, *P* = 0.016). Compared with the lowest quartile, the highest quartile was associated with higher odds of composite glaucoma (OR = 1.66, 95% CI: 1.12–2.46, *P* = 0.026), and the trend test was statistically significant (*P* for trend = 0.037). Findings for self-reported glaucoma were consistent in direction and statistically significant, whereas examination-based glaucoma showed a consistent direction but weaker statistical evidence. The association remained statistically significant after excluding participants with WBC counts >10 × 10^9^/L. After false discovery rate correction, AISI and SIRI remained significantly associated with composite glaucoma. In the clinical sample, AISI levels were higher in patients with primary glaucoma than in the control groups.

**Conclusions:**

Higher AISI was associated with higher odds of glaucoma among adults. As a blood-cell-derived inflammatory index based on routine peripheral blood counts, AISI may provide complementary evidence linking systemic inflammatory burden with glaucoma status. The clinical validation sample further supported this association.

## Introduction

Glaucoma is one of the leading causes of irreversible blindness worldwide, and its disease burden continues to increase (1, 2). It has been estimated that approximately 4.22 million adults in the United States had glaucoma in 2022, including about 1.49 million with glaucoma-related visual impairment (3). Glaucoma is characterized by progressive optic nerve damage and visual field loss, and it often has an insidious course with nonspecific symptoms in the early stage; as a result, some patients already have irreversible visual impairment at the time of diagnosis (4). Therefore, early identification of high-risk individuals and targeted screening are important for glaucoma prevention and control. In recent years, population-based studies using the National Health and Nutrition Examination Survey (NHANES) have been applied to evaluate glaucoma and its associations with systemic health factors, providing epidemiologic evidence for exploring systemic factors related to glaucoma (5–7).

Accumulating evidence suggests that neuroinflammation and immune dysregulation play important roles in the pathogenesis of glaucoma (8, 9). Chronic inflammation may contribute to the development and progression of glaucoma through multiple pathways, including oxidative stress, vascular dysfunction, glial cell activation, and trabecular meshwork injury (9, 10). Composite inflammatory indices derived from peripheral blood cell counts have received increasing attention because they are inexpensive, readily available, and have been increasingly investigated for risk stratification and prognostic assessment. These indices combine neutrophil, monocyte, lymphocyte, and platelet counts through products or ratios to reflect systemic inflammatory and immune status. The aggregate index of systemic inflammation (AISI) is one such index and is calculated from peripheral neutrophil, monocyte, platelet, and lymphocyte counts (11). By integrating information from multiple blood cell types, AISI may provide a broader measure of systemic inflammatory status than a single cell count or simple cell ratio.

As a composite inflammatory index that has received increasing attention in recent years, AISI has shown potential value for risk stratification or prognostic assessment in a variety of systemic diseases (12, 13). In ophthalmology, other composite inflammatory indices, such as the systemic immune-inflammation index (SII) and the systemic inflammatory response index (SIRI), have been reported to be associated with glaucoma and other ocular diseases (14–16). However, direct evidence on the association between AISI and glaucoma is still lacking, particularly in large, nationally representative populations.

Therefore, using NHANES data, we investigated the association between AISI and glaucoma among adults aged 40 years and older. We focused on whether AISI, a peripheral blood-cell-derived inflammatory index, was associated with glaucoma across different outcome definitions and whether this association differed from related inflammatory indices. In addition, we used a hospital-based clinical sample to validate the main findings.

## Materials and methods

### Study population

This study was a cross-sectional analysis based on data from the National Health and Nutrition Examination Survey (NHANES), a program conducted by the US Centers for Disease Control and Prevention. NHANES uses a complex, stratified, multistage probability sampling design to collect health-related information from the noninstitutionalized US population through household interviews, mobile examination center (MEC) examinations, and laboratory testing. We included NHANES 2005–2008 data because this period provided both glaucoma-related questionnaire data and retinal photographs used for the examination-based glaucoma definition.

**Figure 1:**
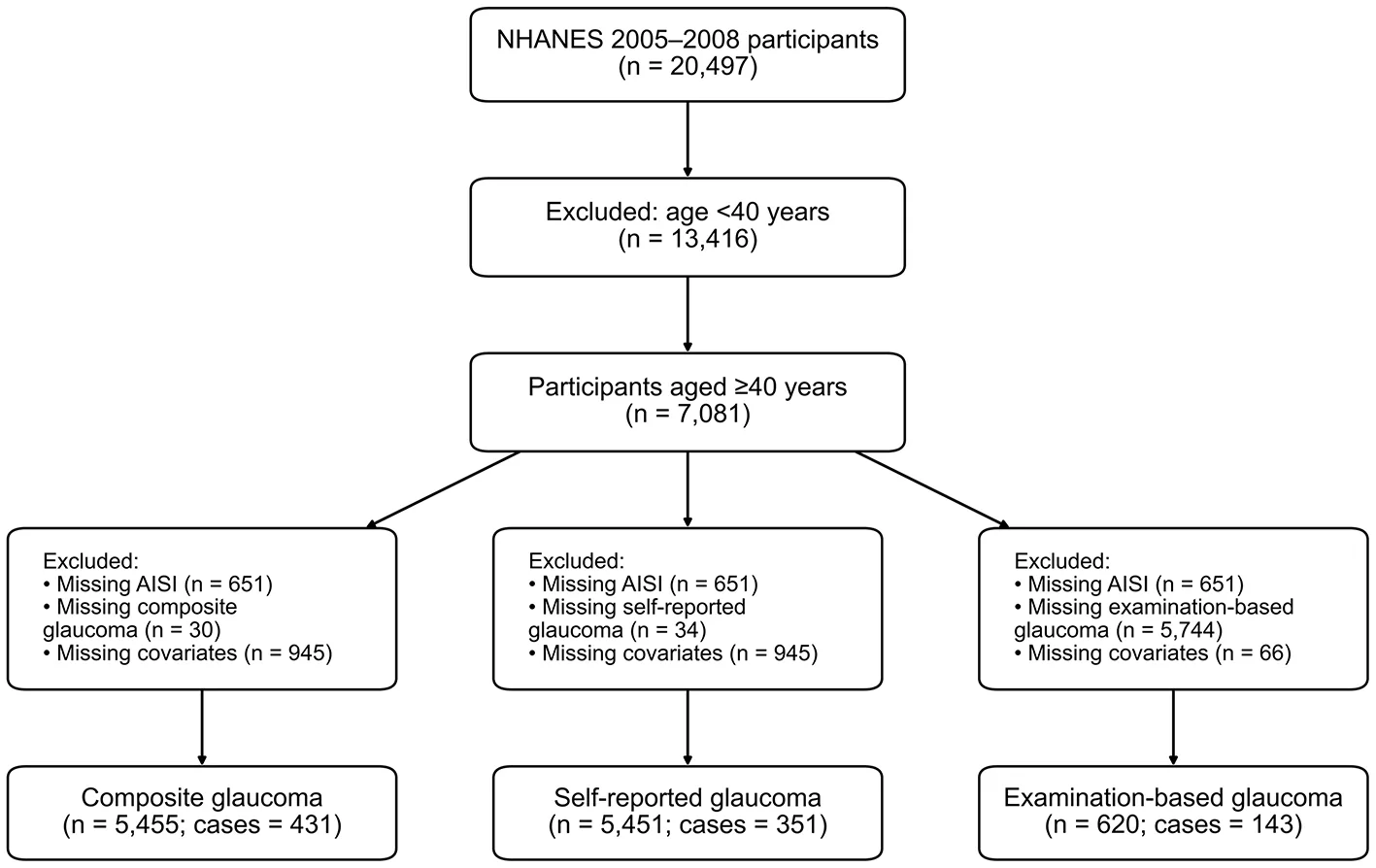
Flow diagram of participant selection for the main and sensitivity analyses.

A total of 20,497 participants were included in the two original survey cycles. After excluding 13,416 participants aged < 40 years, 7,081 participants remained. Among adults aged ≥40 years, the primary analysis further excluded participants with missing data on AISI, composite glaucoma status, or covariates, leaving 5,455 participants for analysis, including 431 glaucoma cases. Sensitivity analyses were performed using self-reported glaucoma and examination-based glaucoma as outcomes. The self-reported glaucoma analysis included 5,451 participants, including 351 cases, and the examination-based glaucoma analysis included 620 participants, including 143 cases. The participant selection process is shown in Figure 1.

### Exposure variables

The primary exposure was the aggregate index of systemic inflammation (AISI). AISI was calculated using peripheral blood neutrophil, monocyte, platelet, and lymphocyte counts according to the following formula: AISI = neutrophil count × monocyte count × platelet count/lymphocyte count (11). In the analyses, log_2_-transformed AISI was analyzed as a continuous variable and was also categorized into quartiles, with the lowest quartile used as the reference group. In the continuous models, the effect estimate was interpreted per doubling of AISI.

To compare AISI with related blood-cell-derived inflammatory indices, we also calculated the systemic inflammatory response index (SIRI), systemic immune-inflammation index (SII), neutrophil-to-lymphocyte ratio (NLR), and platelet-to-lymphocyte ratio (PLR). SIRI was calculated as neutrophil count × monocyte count/lymphocyte count (17), and SII as platelet count × neutrophil count/lymphocyte count (18). NLR and PLR were calculated as neutrophil count/lymphocyte count and platelet count/lymphocyte count, respectively (11).

### Outcome variable

The outcome was glaucoma. Self-reported glaucoma was defined according to the relevant question in the vision and eye health questionnaire. Participants who answered “yes” were classified as having self-reported glaucoma, whereas those who answered “no” were classified as not having self-reported glaucoma. Responses of “don't know,” “refused,” and missing values were treated as missing. Examination-based glaucoma was defined according to grading results from bilateral retinal photographs; participants were classified as having examination-based glaucoma if either eye was graded as probable glaucoma or definite glaucoma. Composite glaucoma was defined as the presence of either self-reported glaucoma or examination-based glaucoma. Because self-reported glaucoma and examination-based glaucoma do not completely overlap in the NHANES 2005–2008 population (19), we used composite glaucoma as the primary outcome and performed sensitivity analyses using self-reported glaucoma and examination-based glaucoma separately.

### Covariates

Based on previous literature and data availability, the following potential confounders were included: age, sex, race/ethnicity, education level, poverty income ratio (PIR), body mass index (BMI), smoking status, drinking status, hypertension, and diabetes. Age, PIR, and BMI were treated as continuous variables, whereas the remaining covariates were treated as categorical variables. Smoking status was categorized as never, former, or current smoking according to whether the participant had smoked at least 100 cigarettes in their lifetime and their current smoking status. Drinking status was categorized as drinker or non-drinker. Hypertension was defined as a previous physician diagnosis of hypertension, or a mean systolic blood pressure ≥140 mmHg, or a mean diastolic blood pressure ≥90 mmHg; mean systolic and diastolic blood pressures were calculated as the averages of up to four blood pressure measurements. Diabetes was defined as a previous physician diagnosis of diabetes or glycated hemoglobin (HbA1c) ≥6.5%.

### Statistical analysis

All analyses accounted for the complex NHANES sampling design and were weighted. Because the exposure variable and some covariates were derived from MEC examination and laboratory data, the 4-year MEC examination weight (WTMEC4YR) was used. This weight was calculated from the 2-year MEC examination weights for the 2005–2006 and 2007–2008 cycles according to NHANES recommendations. Continuous variables are presented as weighted means ± standard deviations, and categorical variables are presented as weighted percentages. Baseline characteristics were compared across AISI quartiles using weighted linear regression for continuous variables and the Rao–Scott χ^2^ test for categorical variables.

Weighted multivariable logistic regression analyses were performed to examine the association between AISI and glaucoma. Model 1 was unadjusted. Model 2 was adjusted for age, sex, and race/ethnicity. Model 3 was further adjusted for education level, PIR, BMI, smoking status, drinking status, hypertension, and diabetes. AISI was analyzed as both a continuous variable and a quartile-based categorical variable, and a trend test was performed. For the trend test, AISI quartiles were entered into the model as an ordinal variable. Restricted cubic spline models were used to assess the exposure–response relationship between AISI and the prevalence of composite glaucoma and to test for overall and nonlinear associations. Subgroup analyses were conducted according to age, sex, BMI, hypertension, and diabetes, and between-group heterogeneity was assessed using interaction terms. The primary analysis used composite glaucoma as the outcome, and sensitivity analyses repeated the weighted multivariable logistic regression models using self-reported glaucoma and examination-based glaucoma as outcomes. The main model was also repeated after excluding participants with AISI values below the 1st percentile or above the 99th percentile. In a separate sensitivity analysis, participants with a white blood cell (WBC) count >10 × 10^9^/L were excluded to reduce the potential influence of acute inflammatory conditions. Associations of AISI, SIRI, SII, NLR, and PLR with composite glaucoma were estimated using the same fully adjusted survey-weighted logistic regression model. *P*-values were obtained from design-adjusted Wald tests, and the Benjamini–Hochberg procedure was used to control the false discovery rate across the five inflammatory indices.

### Clinical validation sample

To validate the findings from the NHANES analysis, we further included a single-center retrospective hospital-based clinical sample. All clinical participants were recruited from the Department of Ophthalmology and the Health Examination Center of The People's Hospital of the Qiandongnan Miao and Dong Autonomous Prefecture between January 2023 and May 2026. The clinical sample included patients with primary glaucoma, cataract controls, and non-glaucoma and non-cataract health examination controls.

Inclusion criteria were as follows: (1) age ≥40 years; (2) complete clinical and laboratory data, including peripheral blood cell counts, anthropometric measurements, and major clinical covariates; and (3) a clearly defined clinical group based on ophthalmic examination.

Exclusion criteria were as follows: (1) secondary glaucoma or other ocular diseases that could substantially affect group classification; (2) acute attacks of primary acute angle-closure glaucoma; and (3) missing key laboratory or clinical data.

All clinical group assignments were determined by ophthalmologists based on ophthalmic examination. Primary glaucoma was diagnosed according to intraocular pressure measurement, gonioscopy, optic disc or retinal nerve fiber layer assessment, and visual field testing, as available in routine clinical practice. After consecutive screening and exclusion of secondary glaucoma and acute attack cases, the primary glaucoma group included primary open-angle glaucoma, chronic primary angle-closure glaucoma, and primary acute angle-closure glaucoma outside the acute attack phase. Cataract was defined as age-related lens opacity confirmed by slit-lamp biomicroscopy; cataract controls were patients meeting this definition without evidence of glaucoma. Non-glaucoma and non-cataract health examination controls were defined as health examination participants without evidence of glaucoma or cataract on ophthalmic examination.

Peripheral blood cell counts were obtained from routine automated hematology analysis and included neutrophil, lymphocyte, monocyte, and platelet counts. AISI in the clinical sample was calculated using the same formula as in the NHANES analysis.

In the clinical sample, continuous variables are presented as means ± standard deviations or medians with interquartile ranges, as appropriate, and categorical variables are presented as counts and percentages. Between-group comparisons were performed using one-way analysis of variance, the Kruskal–Wallis test, or the χ^2^ test, as appropriate. Logistic regression was used to compare the association of AISI with primary glaucoma status using cataract controls and non-glaucoma and non-cataract health examination controls as comparators. Model 1 was unadjusted, Model 2 was adjusted for age and sex, and Model 3 was further adjusted for BMI, hypertension, diabetes, smoking history, and drinking history. The fully adjusted model was repeated after excluding participants with WBC counts >10 × 10^9^/L. The clinical sample was used to validate the direction of the association between AISI and glaucoma status in a clinical population.

Statistical analyses were performed using R software (version 4.5.3; R Foundation for Statistical Computing, Vienna, Austria) with the survey package. All tests were two-sided, and *P* < 0.05 was considered statistically significant.

## Results

### Characteristics of the study population

The primary analysis included 5,455 participants aged ≥40 years, of whom 431 had composite glaucoma. The weighted mean age of the study population was 56.7 ± 11.9 years, 47.4% were men, and the weighted prevalence of composite glaucoma was 6.0%.

After participants were grouped according to AISI quartiles, 1,364, 1,364, 1,363, and 1,364 participants were included in Q1, Q2, Q3, and Q4, respectively. With increasing AISI levels, BMI, the proportion of participants with hypertension, and the proportion of current smokers generally increased. The weighted prevalence of composite glaucoma was 4.9% in Q1 and 7.5% in Q4. Baseline characteristics are shown in Table 1.

**Table 1:** Weighted baseline characteristics of participants according to quartiles of AISI.

Characteristic	Overall	Q1	Q2	Q3	Q4	*P*-value
		**(12.2–179.0)**	**(179.0–270.3)**	**(270.6–415.6)**	**(416.0–4608.0)**	
N	5,455	1,364	1,364	1,363	1,364	
Age, years	56.71 ± 11.94	56.42 ± 11.34	56.20 ± 11.40	56.67 ± 11.94	57.44 ± 12.84	0.330
**Sex, %**						0.518
Male	47.4	46.4	45.9	48.6	48.5	
Female	52.6	53.6	54.1	51.4	51.5	
**Race/ethnicity, %**						< 0.001
Mexican American	5.4	6.0	5.8	6.0	3.9	
Other Hispanic	3.0	3.9	3.4	2.9	1.9	
Non-Hispanic white	77.5	65.7	75.9	80.0	85.8	
Non-Hispanic black	9.6	19.2	9.7	7.1	4.3	
Other Race	4.6	5.1	5.2	4.0	4.2	
**Education level, %**						0.072
< High school	18.2	19.2	16.8	19.9	17.0	
High school/equivalent	26.0	24.8	25.2	24.4	29.1	
>High school	55.8	56.0	57.9	55.7	54.0	
Poverty income ratio	3.29 ± 1.58	3.30 ± 1.60	3.36 ± 1.56	3.29 ± 1.58	3.21 ± 1.57	0.330
Body mass index, kg/m^2^	29.15 ± 6.58	28.36 ± 5.78	29.10 ± 6.22	29.34 ± 6.64	29.63 ± 7.35	< 0.001
**Smoking status, %**						< 0.001
Never	48.9	53.0	52.5	48.7	42.7	
Former	31.1	30.8	30.1	31.1	32.2	
Current	20.0	16.2	17.4	20.2	25.1	
**Drinking status, %**						0.070
No	27.9	29.9	30.0	26.3	26.1	
Yes	72.1	70.1	70.0	73.7	73.9	
**Hypertension, %**						0.021
No	50.1	54.1	52.3	48.3	46.6	
Yes	49.9	45.9	47.7	51.7	53.4	
**Diabetes, %**						0.851
No	85.8	85.4	86.7	85.4	85.6	
Yes	14.2	14.6	13.3	14.6	14.4	
**Composite glaucoma, %**						0.076
No	94.0	95.1	94.3	94.3	92.5	
Yes	6.0	4.9	5.7	5.7	7.5	

Data are presented as weighted mean ± SD for continuous variables and weighted percentages for categorical variables. P-values were derived from weighted linear regression for continuous variables and Rao–Scott χ^2^ tests for categorical variables. Quartiles were based on AISI in the main analysis dataset.

### Association between AISI and composite glaucoma

Overall, higher AISI was associated with higher odds of composite glaucoma. In the unadjusted model, each doubling of AISI was associated with higher odds of composite glaucoma (OR = 1.20, 95% CI: 1.05–1.38, *P* = 0.013). After adjustment for age, sex, and race/ethnicity, this association remained (OR = 1.20, 95% CI: 1.05–1.37, *P* = 0.011). In the fully adjusted model, each doubling of AISI remained associated with higher odds of composite glaucoma (OR = 1.20, 95% CI: 1.05–1.37, *P* = 0.016).

When AISI was analyzed in quartiles, no statistically significant associations were observed for Q2 or Q3 compared with Q1, whereas Q4 was associated with higher odds of composite glaucoma (OR = 1.66, 95% CI: 1.12–2.46, *P* = 0.026). The trend test was also statistically significant (*P* for trend = 0.037). The results are shown in Table 2.

**Table 2:** Weighted associations between AISI and composite glaucoma.

**Exposure**	Model 1		Model 2		Model 3	
	**OR (95% CI)**	***P*** **-value**	**OR (95% CI)**	***P*** **-value**	**OR (95% CI)**	***P*** **-value**
AISI	1.20 (1.05, 1.38)	0.013	1.20 (1.05, 1.37)	0.011	1.20 (1.05, 1.37)	0.016
**AISI quartiles**						
Q1 (reference)	Reference		Reference		Reference	
Q2	1.17 (0.81, 1.68)	0.413	1.30 (0.92, 1.85)	0.153	1.31 (0.93, 1.84)	0.150
Q3	1.18 (0.87, 1.59)	0.296	1.29 (0.96, 1.73)	0.106	1.27 (0.94, 1.72)	0.137
Q4	1.58 (1.09, 2.27)	0.021	1.65 (1.12, 2.44)	0.020	1.66 (1.12, 2.46)	0.026
*P* for trend	0.029		0.030		0.037	

Model 1: unadjusted. Model 2: adjusted for age, sex, and race/ethnicity. Model 3: further adjusted for education level, poverty income ratio, body mass index, smoking status, alcohol drinking, hypertension, and diabetes. For continuous analysis, AISI was log_2_-transformed; therefore, the OR represents the association per doubling of AISI. Results are presented as odds ratios (ORs) with 95% confidence intervals (CIs).

### Restricted cubic spline analysis

**Figure 2:**
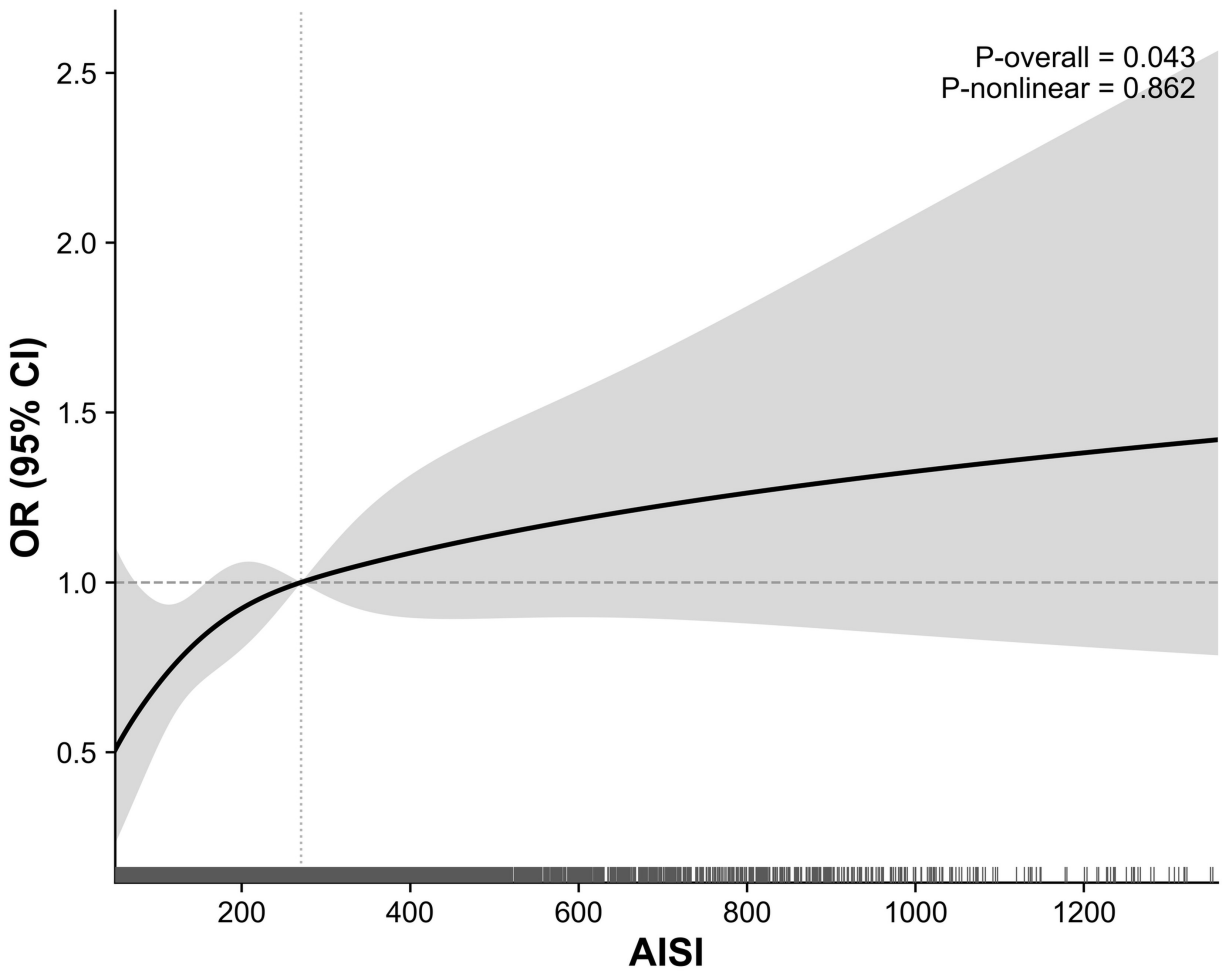
Restricted cubic spline analysis of the association between AISI and composite glaucoma. The solid line represents the adjusted odds ratio (OR), and the shaded area represents the 95% confidence interval (CI). *P*-overall and *P*-nonlinear are shown in the figure.

Restricted cubic spline analysis showed that, in the fully adjusted model, AISI was significantly associated with the prevalence of composite glaucoma overall (*P*-overall = 0.043), whereas no significant nonlinear association was detected (*P*-nonlinear = 0.862). Overall, the odds of composite glaucoma increased with higher AISI levels (Figure 2).

### Subgroup analyses

**Figure 3:**
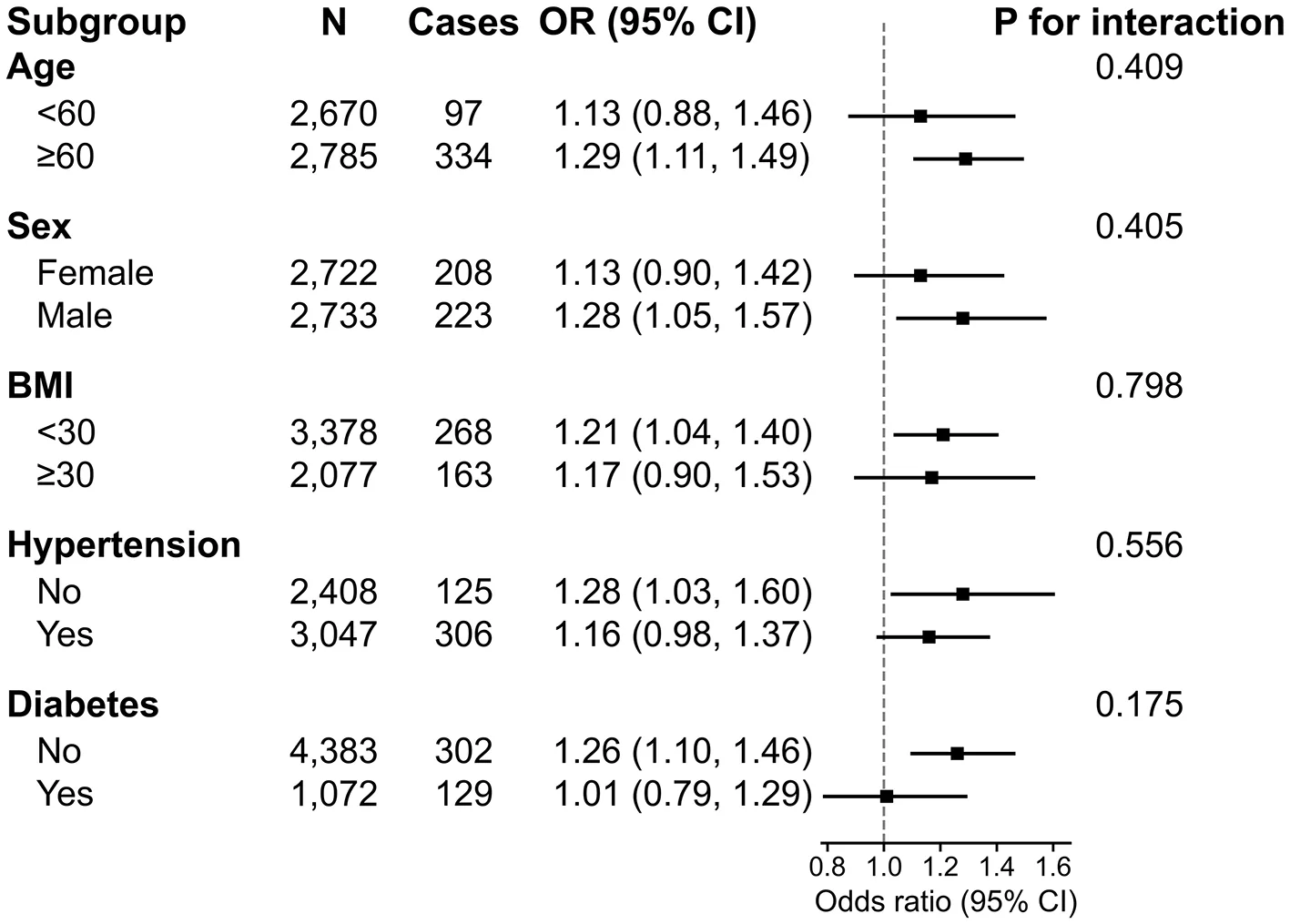
Subgroup analyses of the association between AISI and composite glaucoma. Odds ratios (ORs) and 95% confidence intervals (CIs) are shown for each subgroup. The dashed vertical line indicates an OR of 1.0.

Subgroup analyses showed that the association between AISI and composite glaucoma was generally consistent across strata defined by age, sex, BMI, hypertension, and diabetes. Point estimates were relatively higher among participants aged ≥60 years, men, those with BMI < 30 kg/m^2^, those without hypertension, and those without diabetes, and some subgroup-specific associations reached statistical significance. However, none of the interaction tests reached statistical significance (all *P* for interaction >0.05), indicating no clear evidence of between-group heterogeneity (Figure 3).

### Sensitivity analyses

Sensitivity analyses showed generally directionally consistent associations across different glaucoma definitions. When the analysis was repeated using self-reported glaucoma as the outcome, the results were directionally consistent with those of the primary analysis. In the fully adjusted model, each doubling of AISI was associated with higher odds of self-reported glaucoma (OR = 1.19, 95% CI: 1.04–1.36, *P* = 0.020). In the quartile-based analysis, Q4 was associated with higher odds of self-reported glaucoma compared with Q1 (OR = 1.63, 95% CI: 1.08–2.47, *P* = 0.038), and the trend test was also statistically significant (*P* for trend = 0.047).

When the analysis was repeated using examination-based glaucoma as the outcome, the direction of association was consistent with that of the primary analysis, but the association did not reach statistical significance in the fully adjusted model (OR = 1.30, 95% CI: 0.99–1.71, *P* = 0.080). In the quartile-based analysis, Q2, Q3, and Q4 were not significantly associated with examination-based glaucoma compared with Q1, and the trend test was also not statistically significant (*P* for trend = 0.079). The results are shown in Table 3.

**Table 3:** Sensitivity analyses using self-reported glaucoma and examination-based glaucoma as outcomes.

Exposure	Model 1		Model 2		Model 3	
	**OR (95% CI)**	***P*** **-value**	**OR (95% CI)**	***P*** **-value**	**OR (95% CI)**	***P*** **-value**
**Panel A. Self-reported glaucoma**						
15.6-7.2,-1.3498ptAISI	1.20 (1.04, 1.37)	0.015	1.19 (1.05, 1.35)	0.014	1.19 (1.04, 1.36)	0.020
AISI quartiles						
Q1 (reference)	Reference		Reference		Reference	
Q2	1.12 (0.73, 1.73)	0.607	1.25 (0.82, 1.91)	0.320	1.25 (0.83, 1.89)	0.298
Q3	1.17 (0.86, 1.59)	0.319	1.28 (0.93, 1.75)	0.145	1.27 (0.94, 1.73)	0.147
Q4	1.56 (1.07, 2.26)	0.028	1.62 (1.07, 2.43)	0.031	1.63 (1.08, 2.47)	0.038
*P* for trend	0.033		0.038		0.047	
Panel B. Examination-based glaucoma						
AISI	1.51 (1.20, 1.90)	0.002	1.29 (1.00, 1.68)	0.065	1.30 (0.99, 1.71)	0.080
AISI quartiles						
Q1 (reference)	Reference		Reference		Reference	
Q2	1.05 (0.42, 2.59)	0.922	1.09 (0.39, 3.01)	0.870	1.07 (0.39, 2.92)	0.901
Q3	1.41 (0.64, 3.07)	0.401	1.37 (0.57, 3.28)	0.485	1.45 (0.58, 3.66)	0.441
Q4	2.51 (1.19, 5.32)	0.023	1.98 (0.84, 4.68)	0.134	1.98 (0.80, 4.86)	0.162
*P* for trend	0.006		0.061		0.079	

Sensitivity analyses repeated the main weighted logistic regression models using self-reported glaucoma and examination-based glaucoma as outcomes, respectively. Covariate adjustment for Models 1–3 was identical to Table 2. For continuous analysis, AISI was log_2_-transformed; therefore, the OR represents the association per doubling of AISI.

After excluding participants with AISI values below the 1st percentile or above the 99th percentile, the association between AISI and composite glaucoma remained directionally consistent and statistically significant in the fully adjusted model (OR = 1.19, 95% CI: 1.02–1.40, *P* = 0.048). The association also remained statistically significant after excluding participants with WBC counts >10 × 10^9^/L (*N* = 5,013; cases = 399; OR = 1.17, 95% CI: 1.01–1.36, *P* = 0.035). In comparative analyses of related blood-cell-derived inflammatory indices, AISI and SIRI were both positively associated with composite glaucoma after full adjustment and remained statistically significant after false discovery rate correction (AISI: OR = 1.20, 95% CI: 1.05–1.37, *P* = 0.016, FDR *q* = 0.044; SIRI: OR = 1.23, 95% CI: 1.06–1.44, *P* = 0.017, FDR *q* = 0.044). In contrast, SII, NLR, and PLR did not remain statistically significant after correction for multiple comparisons. These results are shown in Table 4.

**Table 4:** Sensitivity and comparative analyses of AISI and related blood-cell-derived inflammatory indices in relation to composite glaucoma.

Analysis or index	N	Cases	OR per doubling (95% CI)	*P*-value (FDR q)
Panel A. Sensitivity analyses				
Excluding AISI values < 1st or >99th percentile	5,345	419	1.19 (1.02, 1.40)	0.048
Excluding participants with WBC >10 × 10^9^/L	5,013	399	1.17 (1.01, 1.36)	0.035
Panel B. Comparative analyses of related blood-cell-derived inflammatory indices				
AISI	5,455	431	1.20 (1.05, 1.37)	0.016 (0.044)
SIRI	5,455	431	1.23 (1.06, 1.44)	0.017 (0.044)
SII	5,455	431	1.18 (1.00, 1.40)	0.072 (0.119)
NLR	5,455	431	1.19 (0.98, 1.45)	0.102 (0.127)
PLR	5,455	431	1.11 (0.91, 1.35)	0.322 (0.322)

Data are presented as odds ratios (ORs) with 95% confidence intervals (CIs). Panel A repeated the fully adjusted AISI model after excluding participants with extreme AISI values and after excluding participants with WBC >10 × 10^9^/L, respectively. Panel B shows fully adjusted models for related blood-cell-derived inflammatory indices. In Panel B, FDR q values are shown in parentheses after P-values. All inflammatory indices were log_2_-transformed; therefore, the OR represents the association per doubling of each index. All models were adjusted for age, sex, race/ethnicity, education level, poverty income ratio, body mass index, smoking status, alcohol drinking, hypertension, and diabetes. False discovery rate correction was applied across AISI, SIRI, SII, NLR, and PLR using the Benjamini–Hochberg method. AISI, aggregate index of systemic inflammation; SIRI, systemic inflammatory response index; SII, systemic immune-inflammation index; NLR, neutrophil-to-lymphocyte ratio; PLR, platelet-to-lymphocyte ratio; FDR, false discovery rate.

### Clinical data validation

The hospital-based clinical sample included 1,216 participants: 316 patients with primary glaucoma, 300 cataract controls, and 600 non-glaucoma and non-cataract health examination controls. Baseline characteristics of the clinical sample are shown in Table 5. AISI levels were higher in the primary glaucoma group than in the control groups.

**Table 5:** Baseline characteristics of the hospital-based clinical validation sample.

Characteristic	Primary glaucoma	Cataract controls	Health examination controls	*P*-value
N	316	300	600	
Age, years	64.74 ± 11.72	69.78 ± 11.38	53.09 ± 7.48	< 0.001
**Sex, %**				< 0.001
Male	131 (41.5)	115 (38.3)	342 (57.0)	
Female	185 (58.5)	185 (61.7)	258 (43.0)	
Body mass index, kg/m^2^	23.67 ± 3.67	23.39 ± 3.55	25.15 ± 3.39	< 0.001
**Smoking history, %**				0.685
No	260 (82.3)	252 (84.0)	490 (81.7)	
Yes	56 (17.7)	48 (16.0)	110 (18.3)	
**Drinking history, %**				0.737
No	213 (67.4)	209 (69.7)	403 (67.2)	
Yes	103 (32.6)	91 (30.3)	197 (32.8)	
**Hypertension, %**				< 0.001
No	179 (56.6)	171 (57.0)	441 (73.5)	
Yes	137 (43.4)	129 (43.0)	159 (26.5)	
**Diabetes, %**				0.466
No	279 (88.3)	273 (91.0)	543 (90.5)	
Yes	37 (11.7)	27 (9.0)	57 (9.5)	
WBC, × 10^9^/L	6.82 (5.76, 8.23)	5.95 (4.94, 7.06)	6.33 (5.36, 7.47)	< 0.001
Neutrophil count, × 10^9^/L	4.38 (3.37, 5.74)	3.42 (2.72, 4.47)	3.56 (2.94, 4.49)	< 0.001
Lymphocyte count, × 10^9^/L	1.69 (1.34, 2.04)	1.78 (1.31, 2.18)	2.01 (1.68, 2.46)	< 0.001
Monocyte count, × 10^9^/L	0.40 (0.32, 0.53)	0.38 (0.31, 0.48)	0.36 (0.29, 0.43)	< 0.001
Platelet count, × 10^9^/L	213.00 (177.75, 267.00)	200.00 (161.75, 244.25)	214.00 (178.00, 250.00)	< 0.001
AISI	224.93 (141.99, 377.57)	151.06 (89.65, 261.44)	133.52 (89.20, 198.75)	< 0.001
**WBC** **>10** **×10**^**9**^**/L, %**				< 0.001
No	289 (91.5)	295 (98.3)	583 (97.2)	
Yes	27 (8.5)	5 (1.7)	17 (2.8)	

Data are presented as mean ± SD, median (interquartile range), or n (%), as appropriate. P-values were derived from one-way analysis of variance for normally distributed continuous variables, Kruskal–Wallis tests for skewed continuous variables, and χ^2^ tests for categorical variables. AISI, aggregate index of systemic inflammation; WBC, white blood cell count.

Logistic regression analyses further showed that higher AISI was associated with clinically diagnosed primary glaucoma. The association remained consistent in the fully adjusted models when primary glaucoma was compared with either non-glaucoma and non-cataract health examination controls or cataract controls. The results were not materially changed after excluding participants with WBC counts >10 × 10^9^/L. The clinical validation results are shown in Table 6.

**Table 6:** Association between AISI and primary glaucoma in the hospital-based clinical validation sample.

**Analysis sample**	Model	*N*	OR per doubling (95% CI)	*P*-value
Panel A. Primary glaucoma vs. health examination controls				
Full sample	Model 1	916	2.25 (1.92, 2.64)	< 0.001
	Model 2	916	2.36 (1.96, 2.84)	< 0.001
	Model 3	916	2.40 (1.99, 2.90)	< 0.001
WBC ≤ 10 × 10^9^/L	Model 3	872	2.33 (1.89, 2.86)	< 0.001
Panel B. Primary glaucoma vs. cataract controls				
Full sample	Model 1	616	1.53 (1.32, 1.78)	< 0.001
	Model 2	616	1.57 (1.35, 1.83)	< 0.001
	Model 3	616	1.58 (1.35, 1.84)	< 0.001
WBC ≤ 10 × 10^9^/L	Model 3	584	1.54 (1.31, 1.82)	< 0.001

Model 1 was unadjusted. Model 2 was adjusted for age and sex. Model 3 was further adjusted for body mass index, hypertension, diabetes, smoking history, and drinking history. AISI was log_2_-transformed; therefore, the OR represents the association per doubling of AISI. The WBC sensitivity analysis excluded participants with WBC counts >10 × 10^9^/L. AISI, aggregate index of systemic inflammation; CI, confidence interval; OR, odds ratio; WBC, white blood cell count.

## Discussion

In this population-based analysis of NHANES data, we evaluated the association between AISI and glaucoma prevalence among adults aged 40 years and older. We found that, after adjustment for multiple potential confounders, higher AISI was associated with higher odds of composite glaucoma. Compared with the lowest quartile, the highest quartile was associated with significantly higher odds of composite glaucoma, and the trend test was statistically significant. Sensitivity analyses showed that the association between AISI and glaucoma was directionally consistent with the primary analysis when self-reported glaucoma was used as the outcome. When examination-based glaucoma was used as the outcome, the direction of association remained consistent, although statistical significance was not reached. The main findings remained consistent after excluding participants with extreme AISI values and after excluding participants with elevated WBC counts. In the hospital-based clinical validation sample, AISI levels were higher in patients with primary glaucoma than in the control groups, and multivariable analyses supported the same direction of association. No significant interaction was observed in subgroup analyses. Overall, these findings support a positive association between AISI and glaucoma status, while also indicating that the strength of statistical evidence varies according to glaucoma definition and sample size.

Our findings are broadly consistent with previous NHANES studies and other population-based studies of inflammation-related exposures. A recent NHANES study reported that a higher dietary inflammatory index (DII) was positively associated with glaucoma prevalence, suggesting that a pro-inflammatory exposure profile may be linked to a higher prevalence of glaucoma (20). However, DII reflects diet-related inflammatory potential, whereas AISI represents a peripheral blood-cell-derived inflammatory profile. Regarding composite inflammatory indices, Li et al., using the same NHANES 2005–2008 dataset, reported that higher SIRI was positively associated with glaucoma, and the effect size in the high-level group was broadly comparable to the AISI effect observed in our study (14). In addition, a population-based study using broader ocular disease outcomes suggested that systemic inflammatory biomarkers such as SII and SIRI were associated with multiple eye diseases, including glaucoma (15). A recent study combining NHANES and hospital-based clinical data also reported that AISI and related blood-cell-derived inflammatory indices were associated with cataract-related clinical status (21). In the present hospital-based validation sample, AISI was higher in patients with primary glaucoma than in health examination controls without glaucoma or cataract. This direction remained consistent when cataract controls were used as the comparator, suggesting that the finding was not solely attributable to cataract-related inflammation. Compared with previous studies, the present study focused on AISI. In our comparative analyses, AISI and SIRI remained significantly associated with composite glaucoma after false discovery rate correction, whereas SII, NLR, and PLR did not. The association also persisted after excluding participants with WBC counts >10 × 10^9^/L, which reduces the likelihood that the finding was mainly driven by marked leukocytosis or potential acute inflammatory conditions. Because AISI integrates neutrophil, monocyte, platelet, and lymphocyte information, it may reflect a peripheral inflammatory profile that is not fully captured by single cell-ratio indices. Accordingly, the present study extends prior work by evaluating AISI specifically, comparing it with related inflammatory indices derived from peripheral blood cell counts, and examining the association across complementary glaucoma definitions and a hospital-based clinical validation sample.

From a mechanistic perspective, the association between AISI and glaucoma prevalence is biologically plausible. Previous studies have shown that neuroinflammation, immune dysregulation, and glial cell activation are involved in multiple stages of glaucomatous optic nerve injury and may interact with mitochondrial dysfunction, oxidative stress, and neurodegeneration in a self-reinforcing pathological cycle (8, 10, 22, 23). Activated immune cells and glial cells can release pro-inflammatory mediators such as TNF-α and IL-1β, thereby amplifying neuroinflammatory responses and promoting retinal ganglion cell injury (24, 25). Previous studies have also suggested that glaucoma-related neuroinflammation is closely associated with retinal microglial activation, inflammatory cytokine release, and activation of injury-related signaling pathways. In an ischemia-reperfusion model, inhibition of the cGAS-STING pathway attenuated neuroinflammation-mediated retinal ganglion cell death (26). In addition, oxidative stress and inflammatory responses may reinforce each other, and oxidative injury may promote trabecular meshwork degeneration and activation of inflammation-related pathways, thereby affecting aqueous humor outflow resistance and optic nerve vulnerability (10, 27). AISI integrates neutrophil, monocyte, platelet, and lymphocyte information. Monocytes are related to peripheral myeloid inflammatory activation, whereas platelets participate in inflammatory responses, endothelial function, and microvascular changes. Therefore, AISI may better reflect an integrated peripheral inflammatory state involving both chronic low-grade inflammation and vascular-related changes than single cell-ratio indices (9, 28, 29). Against this biological background, the observed association between higher AISI and higher odds of composite glaucoma is plausible. This also suggests that inflammation-related abnormalities relevant to glaucoma may not be confined to ocular tissues alone, but may also leave detectable signals in peripheral blood.

From the overall pattern of results, the association between AISI and composite glaucoma was most evident in the highest quartile, whereas the middle two quartiles were not statistically significant. In addition, restricted cubic spline analysis showed a significant overall association between AISI and composite glaucoma, but no significant nonlinear association. This may indicate that the relationship between AISI and glaucoma is more apparent at higher levels of inflammatory burden rather than showing a completely uniform linear gradient across the full exposure range. This pattern is similar to that reported in some previous studies of composite inflammatory indices, in which continuous-variable models captured the overall trend, whereas significant differences in categorical analyses were mainly concentrated in the high-exposure group (14). Therefore, we interpret the findings as supporting an overall positive association between AISI and glaucoma, with individuals with higher inflammatory burden likely contributing most to this association.

An additional strength of this study was the use of three outcome definitions: composite glaucoma, self-reported glaucoma, and examination-based glaucoma. For self-reported glaucoma, the direction and statistical significance of the association between AISI and the outcome were largely consistent with those of the primary analysis, which supports the stability of the findings. When examination-based glaucoma was used as the outcome, the association remained in the same direction despite not reaching statistical significance. This pattern may partly reflect the smaller analytic sample and differences in outcome ascertainment, and it should be interpreted as weaker statistical evidence for the examination-based definition. A previous NHANES 2005–2008 study showed that self-reported glaucoma and examination-based glaucoma do not completely overlap, and that prevalence estimates and association analyses may differ according to the outcome definition used (19). In our study, the examination-based analysis included only 620 participants, including 143 cases, and therefore had substantially lower statistical power than the primary analysis and the self-reported glaucoma analysis. Accordingly, the lack of statistical significance in the examination-based analysis more likely indicates limited statistical evidence rather than an opposite direction of association.

Subgroup analyses did not suggest clear heterogeneity. Although the point estimates appeared somewhat higher among older participants, men, and participants without diabetes, none of the interaction tests reached statistical significance. This suggests that there is currently insufficient evidence to support the view that the association between AISI and glaucoma is limited to a specific age group, metabolic subgroup, or sex. In other words, the observed association appears to be more consistent with an overall population-level pattern rather than a result clearly driven by a single subgroup. In the absence of significant interaction, differences in point estimates between subgroups should not be overinterpreted.

This study also has several limitations. First, because of the cross-sectional design of the NHANES analysis and the retrospective design of the hospital-based clinical validation sample, our findings only support an association between AISI and glaucoma status and cannot establish temporal sequence or causality. Second, some outcome misclassification may have occurred. Self-reported glaucoma depends on prior diagnosis awareness, whereas examination-based glaucoma was limited by the retinal imaging and related ophthalmic data available in NHANES and cannot fully replace a comprehensive clinical diagnosis. Third, AISI was measured at a single time point and therefore may not reflect long-term inflammatory exposure. Although the findings remained significant after excluding participants with WBC counts >10 × 10^9^/L, the influence of unmeasured inflammatory conditions, including autoimmune diseases and chronic infections, cannot be fully excluded. Fourth, although we adjusted for multiple potential confounders, residual confounding from medication use and other unmeasured factors remains possible. Fifth, the hospital-based clinical validation sample was derived from a single center in China, and the sample size and available covariates remained limited. In particular, the hospital-based sample did not include NHANES-equivalent race/ethnicity, education, or socioeconomic variables; therefore, the hospital-based analyses should be interpreted as clinical validation of the direction of association rather than full population-level replication. Finally, the NHANES analysis did not allow reliable differentiation by glaucoma subtype or disease severity. The clinical validation sample was designed to assess the overall association and did not prespecify subtype comparisons. The relationship between AISI and individual glaucoma subtypes or disease stages therefore warrants further study.

## Conclusion

In conclusion, higher AISI was associated with higher odds of glaucoma among adults aged 40 years and older in NHANES. The association was evident for composite glaucoma and self-reported glaucoma and was directionally consistent for examination-based glaucoma, although with weaker statistical evidence. The association also persisted after excluding participants with elevated WBC counts. As a routine peripheral blood-cell-derived inflammatory index, AISI may provide complementary evidence linking systemic inflammatory burden with glaucoma status. The hospital-based clinical validation sample further supported this association. Prospective longitudinal studies with standardized ophthalmic assessments are needed to determine whether baseline AISI is associated with incident glaucoma. Future studies should also assess whether AISI provides incremental information for glaucoma risk stratification when considered alongside retinal nerve fiber layer thickness, optic disc parameters, and visual field indices.

## Data Availability

The raw data supporting the conclusions of this article will be made available by the authors, without undue reservation.
